# Do Hormonal Fluctuations in Healthy Women Influence the Somatosensory Temporal Discrimination Threshold?

**DOI:** 10.1002/brb3.70790

**Published:** 2025-08-27

**Authors:** Flavia Aiello, Maria Ilenia De Bartolo, Gina Ferrazzano, Giovanni De Fazio, Giovanni Fabbrini, Antonella Conte, Alfredo Berardelli, Daniele Belvisi

**Affiliations:** ^1^ Department of Human Neurosciences Sapienza University of Rome Rome Italy; ^2^ IRCCS Neuromed Pozzilli (IS) Italy; ^3^ Department of Translational Biomedicine and Neuroscience University of Bari Aldo Moro Bari Italy

## Abstract

**Introduction:**

Preclinical studies and observations in human beings demonstrated that ovarian hormones exert complex, time‐dependent effects on multiple brain regions, including those implicated in temporal sensory discrimination. The present study aims to investigate whether somatosensory temporal discrimination threshold (STDT) varies depending on the hormonal fluctuations in healthy women.

**Methods:**

Twenty‐six young women were enrolled, of whom six were taking contraceptive therapy. In each subject, we assessed STDT, both by the step‐wise measurement (sSTDT) and the randomized one (rSTDT), at the three main phases of the ovarian cycle: menstrual phase, ovulation, and the luteal phase.

**Results:**

The Friedman test did not reveal statistically significant differences among the three time points for either sSTDT (χ^2^ = 0.494, *p* = 0.781) or rSTDT (χ^2^ = 0.838, *p* = 0.658). The Mann–Whitney U test for both sSTDT and rSTDT did not reveal statistically significant differences for each time point between subjects who were taking contraceptive therapy with those who were not (T1_st: U = 50, *Z* = −0.620, *p* = 0.573; T1_r: U = 58, *Z* = −0.126, *p* = 0.929; T2_st: U = 50, *Z* = −0.619, *p* = 0.536; T2_r: U = 56, *Z* = −0.248, *p* = 0.836; T3_st: U = 51, *Z* = −0.557, *p* = 0.614; T3_r: U = 52, *Z* = −0.502, *p* = 0.656).

**Conclusions:**

Our study did not reveal any hormone‐dependent variations in STDT across the phases of the ovarian cycle, supporting the notion that STDT is a stable neurophysiological parameter.

## Graphical abstract

The study documented no statistically significant differences in stepwise somatosensory temporal discrimination threshold or randomized somatosensory temporal discrimination threshold across time points or between subjects using or not using contraceptives. Our study did not reveal any hormone‐dependent variations in somatosensory temporal discrimination threshold (STDT) across the phases of the ovarian cycle, supporting the notion that STDT is a stable neurophysiological parameter.

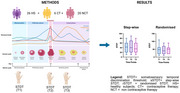

## 1 Introduction

The somatosensory temporal discrimination threshold (STDT) is a neurophysiological test that consists of the identification of the minimum time interval between two consecutive tactile stimuli required to be perceived as distinct in time, enabling the study of sensory integration in normal and pathological conditions (Kaji 2001; Lidsky et al. 1985). Observations in patients with movement disorders have suggested that somatosensory (Conte et al. 2012; Lacruz et al. 1991) and other cortical areas (Lacruz et al. 1991; Pastor et al. 2004), as well as basal ganglia and thalamus, play a critical role in the physiological mechanisms underlying STDT (Conte, Leodori, et al. 2016; Lacruz et al. 1991; Pastor et al. 2004).

Since STDT abnormalities have been consistently reported in patients with dystonia, a movement disorder with a higher frequency observed in women, particularly after menopause (Conte, McGovern, et al. 2017; Velucci et al. 2024), it is important to determine whether in healthy women STDT‐neural processing varies depending on the hormonal fluctuations. Experimental findings have demonstrated that estrogen and progesterone receptors are widely distributed throughout the brain, including key regions involved in sensorimotor processing (Blurton‐Jones and Tuszynski 2002; Brinton et al. 2008; Creutz and Kritzer 2002), and studies in humans have shown that the excitability of cortical areas is influenced by the hormones (Chen and Tseng 2025; Inghilleri et al. 2004; Wang et al. 2014). To this end, in this paper, we investigated STDT in a sample of healthy women, assessing it at three different time points across the menstrual cycle.

## 2 Materials and Methods

### 2.1 Participants

We consecutively enrolled 26 young healthy women (mean age 27.8 ± 2.5) at the Department of Human Neurosciences, Sapienza University of Rome. Inclusion criteria were no neurological or psychiatric diseases, no family history of dystonia, and being in the fertile period. Exclusion criteria were a state of pregnancy or irregular menstrual cycles (a variability of 3 days or greater between the previous 6 months’ menstrual cycles). Six women of the cohort were in regular contraceptive therapy, while the other 20 subjects did not take any type of pharmacological contraception. The study was approved by the Institutional Ethics Committee. All participants gave their written informed consent prior to participating in the study. The study was conducted in accordance with the latest revision of the Declaration of Helsinki.

### 2.2 Somatosensory Temporal Discrimination Threshold

The assessment of STDT was conducted following protocols established in previous studies (Conte, Belvisi, et al. 2016; Conte, Ferrazzano, et al. 2017; Conte, Leodori, et al. 2016). In order to test STDT, we used a constant current stimulator (Digitimer DS7AH). Paired tactile stimuli consisting of square‐wave electrical pulses were delivered to the distal phalanx of the index finger through surface skin electrodes with the subject at rest. Stimulation intensity corresponded to the minimal intensity needed by the subject to perceive 10 out of 10 consecutive stimuli, starting from 2 mA and increasing in 0.5 mA steps. Paired stimuli were delivered starting from an interstimulus interval (ISI) of 0 ms, progressively increased in 10 ms steps, according to the ascending method (step‐wise measurement, sSTDT). The value of sSTDT was considered the first of three consecutive ISIs at which subjects recognized stimuli as temporally separate in an average of three STDT trials. We also investigated STDT according to the randomized method: paired stimuli were delivered with a randomized ISI (randomized measurement, rSTDT), rSTDT was considered the minimum ISI needed to perceive the two stimuli as distinct in the temporal domain, and confirmed three out of three trials.

### 2.3 Experimental Session

Each subject underwent three STDT assessments: the first one within 7 days after the onset of the menstruation (T1), when both estrogen and progesterone are low; the second one around the day 14 of the cycle (T2), when the estrogen peak occurred; and the last one around day 21 of the cycle (T3), at the progestin peak (Anckaert et al. 2021).

### 2.4 Statistical Analysis

Statistical analyses were performed using GraphPad Prism (version 9) and IBM SPSS Statistics (version 30). The Shapiro–Wilk test was used to test all variables for normality. To evaluate changes of STDT across the three time points, considering that STDT values were not normally distributed, we applied the Friedman test (Sheldon et al. 1996) for both the step‐wise measurement (sSTDT) and the randomized measurement (rSTDT). Additionally, to evaluate possible variations in STDT due to contraceptive therapy, we applied a Mann–Whitney U test to both sSTDT and rSTDT, comparing subjects who were taking contraceptive therapy with those who were not.

## 3 Results

**FIGURE 1:**
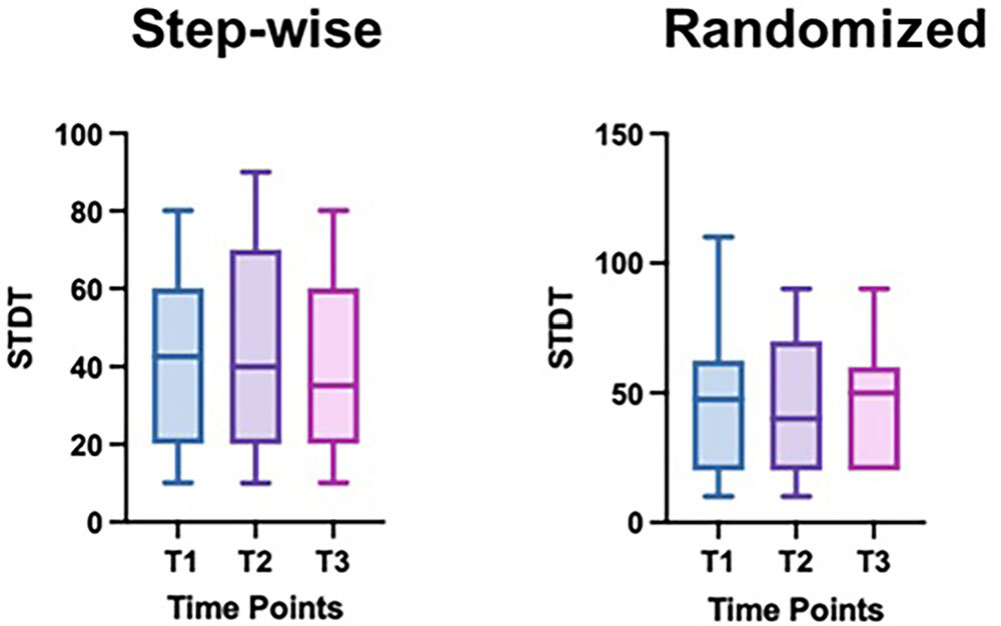
STDT at various time points of the ovarian cycle. T1 = by the day 7 after the onset of menstruation; T2 = day 14 ± 2 of the cycle; T3 = day 21 ± 2 of the cycle. STDT, somatosensory temporal discrimination threshold.

The Friedman test did not reveal statistically significant differences among the three time points for either sSTDT (χ^2^ = 0.494, *p* = 0.781) or rSTDT (χ^2^ = 0.838, *p* = 0.658). Furthermore, the Friedman test did not show any differences in stimulation intensity among the different timepoints (χ^2^ = 1.849, *p* = 0.397). The Mann–Whitney U test for both sSTDT and rSTDT did not reveal statistically significant differences for each time point between subjects who were taking contraceptive therapy with those who were not (T1_st: U = 50, *Z* = −0.620, *p* = 0.573; T1_c: U = 58, *Z* = −0.126, *p* = 0.929; T2_st: U = 50, *Z* = −0.619, *p* = 0.536; T2_c: U = 56, *Z* = −0.248, *p* = 0.836; T3_st: U = 51, *Z* = −0.557, *p* = 0.614; T3_c: U = 52, *Z* = −0.502, *p* = 0.656). Also, stimulation intensity did not differ between groups at various time points (T1: U = 57, *Z* = −0.183, *p* = 0.882; T2: U = 31.5, *Z* = −1.742, *p* = 0.083; T3: U = 58.5, *Z* = −0.092, *p* = 0.929). (See Table 1 and Figure 1.)

**TABLE 1:** Demographic and experimental data.

			Stimulation Intensity			Dominant hemisphere		Ethnicity
	Age (years)	Ovarian cycle duration (days)	Phase I	Phase II	Phase III	R	L	Caucasian
Whole cohort	27.5 (3.7)^a^	28(2)^a^	4.75 (1.2)^a^	5 (1.2)^a^	4.9 (1.2)^a^	3/26 (11.5%)	23/26 (88.5%)	26/26 (100%)
Subjects no‐contraception	28 (4)^a^	30 (4)^a^	4.75 (1)^a^	5 (1.19)^a^	4.65 (1.28)^a^	3/20 (15%)	17/20 (85%)	20/20 (100%)
Subjects in contraception	27 (0.75)^a^	28 (0)^a^	4.5 (1.375)^a^	4.15 (0.825)^a^	5 (0.675)^a^	0/6 (0%)	6/6 (100%)	6/6 (100%)

Abbreviations: L, left; R, right. ^a^Values are expressed as median (interquartile range).

## 4 Discussion

This study examined whether in healthy women hormonal fluctuations occurring throughout the ovarian cycle influence STDT, in light of preclinical studies and observations in human beings clearly demonstrating that ovarian hormones exert complex, time‐dependent effects on multiple brain regions, including those implicated in temporal sensory discrimination (Blurton‐Jones and Tuszynski 2002; Brinton et al. 2008; Creutz and Kritzer 2002; Fernández et al. 2003; S. S. Smith and Woolley 2004; Weis et al. 2008, Weis et al. 2010).

The main finding of this study is the absence of significant variations in STDT across the different phases of the ovarian cycle. STDT remained stable during the menstrual phase, ovulation, and the luteal phase in our sample of 26 healthy women. Furthermore, no significant differences were observed between participants taking hormonal contraceptives and those with natural cycles. This suggests that cyclic hormonal modulation, or its suppression through contraceptive use, does not substantially influence STDT.

The absence of hormonal influence on STDT in healthy women may be explained by several reasons. Sex‐related differences in STDT have been previously documented, with healthy women (Williams et al. 2015) and unaffected female relatives of dystonia patients (Butler et al. 2015) generally showing higher thresholds than men and a steeper age‐related increase in STDT. However, the hormonal fluctuations occurring during the menstrual cycle may be too subtle to significantly influence STDT, compared to the substantial and stable decline in circulating estrogen levels observed during menopause (Verdonk et al. 2019). To the best of our knowledge, no studies have specifically assessed STDT in cohorts of women affected by gynecological conditions associated with marked fluctuations in sex hormone levels (Neitzke et al. 2025). Nonetheless, some studies have investigated other neurophysiological parameters to explore brain function across the menstrual cycle (Avila‐Varela et al. 2024; M. J. Smith et al. 2002; Weis et al. 2008), evidencing dynamic changes in whole‐brain network activity across different menstrual phases, including alterations in the sensorimotor system (Avila‐Varela et al. 2024). Few lines of evidence documented an effect of sex hormones on the clinical progression and severity in Parkinson's disease (PD) (Bovenzi et al. 2025; Jurado‐Coronel et al. 2018), as well as an alteration of STDT in PD (Conte et al. 2018), but no studies investigated the hormone fluctuations in PD and potential correlations to STDT. Neuroimaging studies documented a modulation of cortico‐limbic and reward‐related circuits (Dubol et al. 2021) by ovarian hormone fluctuations, but there is no consistent neuroimaging evidence implicating sensorimotor substrates of STDT in cycle‐based modulation. A previous study showed the lack of menstrual cycle–related changes also for visual temporal discrimination thresholds (TDT) (McGovern et al. 2017), and the auditory TDT variations depending on the sex hormones fluctuations were not assessed. Our results, therefore, may not depend on the specific modality of stimulus presentation, but are rather related to the specific task required. Variations of other parameters accounting for auditory, visual, and sensory systems were documented during the distinct menstrual phases (Aloufi et al. 2023; Avitabile et al. 2007; Barbosa et al. 2013; Eisner et al. 2004; Friedman and Meares 1978; McFadden et al. 2018; Riley et al. 1999; Tasman et al. 1999). A further hypothesis is that STDT may depend on highly specialized and relatively “hardwired” sensorimotor circuits—such as thalamo‐cortical and basal ganglia loops—that are less prone to short‐term modulation by circulating hormones. Temporal resolution, particularly in the millisecond range as required by STDT tasks, may rely on fast, low‐level processing mechanisms with limited plasticity in response to transient physiological changes. Additionally, the central nervous system may engage compensatory mechanisms to maintain temporal discrimination performance despite hormonal shifts. For example, functional redundancy within sensory pathways, including multiple parallel thalamocortical circuits, could buffer minor hormonal effects, preserving perceptual stability. These homeostatic processes, therefore, may help ensure that essential perceptual functions such as temporal discrimination remain constant even in the face of internal neuroendocrine variability. Hence, STDT gender differences are unlikely to be solely hormone‐driven and may reflect slower, intrinsic mechanisms within the central nervous system—possibly involving GABAergic or glutamatergic transmission—consistent with the known modulatory effects of estrogen and progesterone on these neurotransmitter systems (Hsu and Smith 2003; Schloemer et al. 2020; M. J. Smith et al. 2002; S. S. Smith and Woolley 2004). Finally, species differences in hormone receptor expression may partly explain the negative findings in our study. Most supporting data are indeed derived from rodent models, which may limit their direct applicability to humans.

Hence, STDT gender differences are unlikely to be solely hormone‐driven and may reflect slower, intrinsic mechanisms within the central nervous system—possibly involving GABAergic or glutamatergic transmission—consistent with the known modulatory effects of estrogen and progesterone on these neurotransmitter systems (Hsu and Smith 2003; Schloemer et al. 2020; M. J. Smith et al. 2002; S. S. Smith and Woolley 2004).

This study has several limitations. The first limitation was the lack of objective blood, saliva, or urine measurement of circulating hormonal levels. To the best of our knowledge, there are no studies that have validated calendar‐based phase estimation methods; rather, recent literature suggests to carry out objective assays of circulating hormones (Janse DE Jonge et al. 2019). Nonetheless, in order to best account for the individual hormonal variability, at the time of enrolment, each subject who took part in this study provided the records about their last menstrual cycle as reported by personal phone applications. Only subjects with a regular menstrual cycle in the previous 6 months, defined as a cycle length between 28 and 35 days with a variability of no more than ±2 days, were enrolled. A record of the subsequent cycle was also collected to retrospectively confirm the hormonal phase during which the previous assessments were conducted. However, a counting‐based method does not take into consideration anovulatory or luteal phase defect cycles. Therefore, future studies carrying out blood, urine, or saliva circulating hormones assays in a larger cohort are needed to confirm our results. Second, due to recruitment constraints, the sample was unbalanced with respect to hormonal contraceptive use; therefore, we limited our investigation of potential group differences in STDT measures to an exploratory analysis, restraining our ability to draw strong conclusions about its effects. Lastly, our investigation focused exclusively on healthy women; therefore, the generalizability of these findings to patients with dystonia remains unknown.

In conclusion, our study did not reveal any hormone‐dependent variations in STDT across the phases of the ovarian cycle. This finding supports the notion that STDT is a stable neurophysiological parameter, largely unaffected by environmental influences. Since a previous study showed no significant changes in STDT over an 8‐year follow‐up in dystonic patients (Conte, Ferrazzano, et al. 2017), the STDT cannot be considered a marker of disease progression, and it currently represents an endophenotypic feature in dystonia.

Future studies are necessary, involving larger cohorts of subjects, stratified for contraceptive use and type, which may also include neuroimaging studies with GABA and/or glutamate‐magnetic resonance spectroscopy.

## Author Contributions

**Flavia Aiello**: writing – original draft, investigation, methodology. **Maria Ilenia De Bartolo**: writing – original draft, formal analysis, methodology. **Gina Ferrazzano**: writing – review and editing, conceptualization, supervision. **Giovanni De Fazio**: conceptualization, writing – review and editing, supervision. **Giovanni Fabbrini**: conceptualization, writing – review and editing, supervision. **Antonella Conte**: conceptualization, writing—review and editing, supervision. **Alfredo Berardelli**: conceptualization, writing—review and editing, supervision. **Daniele Belvisi**: conceptualization, writing—review and editing, supervision.

## Disclosure

The authors declare no financial disclosures relevant to the present manuscript. Over the past 12 months, A.C. has received speaker honoraria from Roche, Biogen, Novartis, BMS, Almirall, Merck, and Lundbeck, and research support from Biogen, all unrelated to the content of this manuscript. G.Fe. has received consulting fees and speaker honoraria from Merck Serono, Bristol Myers Squibb, Novartis, Sanofi, and Roche, all unrelated to the content of this manuscript.

## Ethics Statement

The study was carried out in agreement with the Declaration of Helsinki.

## Conflicts of Interest

The authors declare no conflicts of interest.

## Consent

Each subject has expressed written informed consent.

## Peer Review

The peer review history for this article is available at https://publons.com/publon/10.1002/brb3.70790.

## Data Availability

Data are available from the corresponding author on reasonable request.
